# A suspected case of gangrenous blepharitis and pre-septal cellulitis caused by Pseudomonas aeruginosa in a neutropenic patient: a case report and literature review

**DOI:** 10.1186/s12348-025-00526-1

**Published:** 2025-09-24

**Authors:** Ali Alyami, Victor Vermot-Desroches, Yasmine Serrar, Philippe Denis

**Affiliations:** Service d’ophtalmologie, Hôpital Universitaire de la Croix-Rousse, Hospices Civils de Lyon, 103, Grande Rue de la Croix-Rousse, 69317, Lyon, Cedex 04 France

## Purpose

To report a case of gangrenous pre-septal cellulitis caused by Pseudomonas aeruginosa in a patient with febrile neutropenia.

## Introduction

The primary pathogens responsible for eyelid infections often include normal microbiota such as Staphylococcus and Streptococcus species [1, 2]. In this case report, we highlight a situation of pre-septal cellulitis occurring during an episode of septicaemia, with Pseudomonas aeruginosa identified through standard blood culture and sensitivity testing performed upon admission in a patient experiencing febrile neutropenia secondary to Myelodysplastic syndrome showing sensitivity to piperacillin-tazobactam, ceftazidime, and ciprofloxacin.

## Case presentation

A 71-year-old male, previously diagnosed with non-Hodgkin lymphoma and myelodysplastic syndrome, was admitted to the infectious disease unit of our hospital due to suspected infection of a hematoma in the left deltoid muscle, following his fourth dose of the COVID-19 vaccine, with a fever of 38.7 recorded at arrival to the emergency department.

**Fig. 1:**
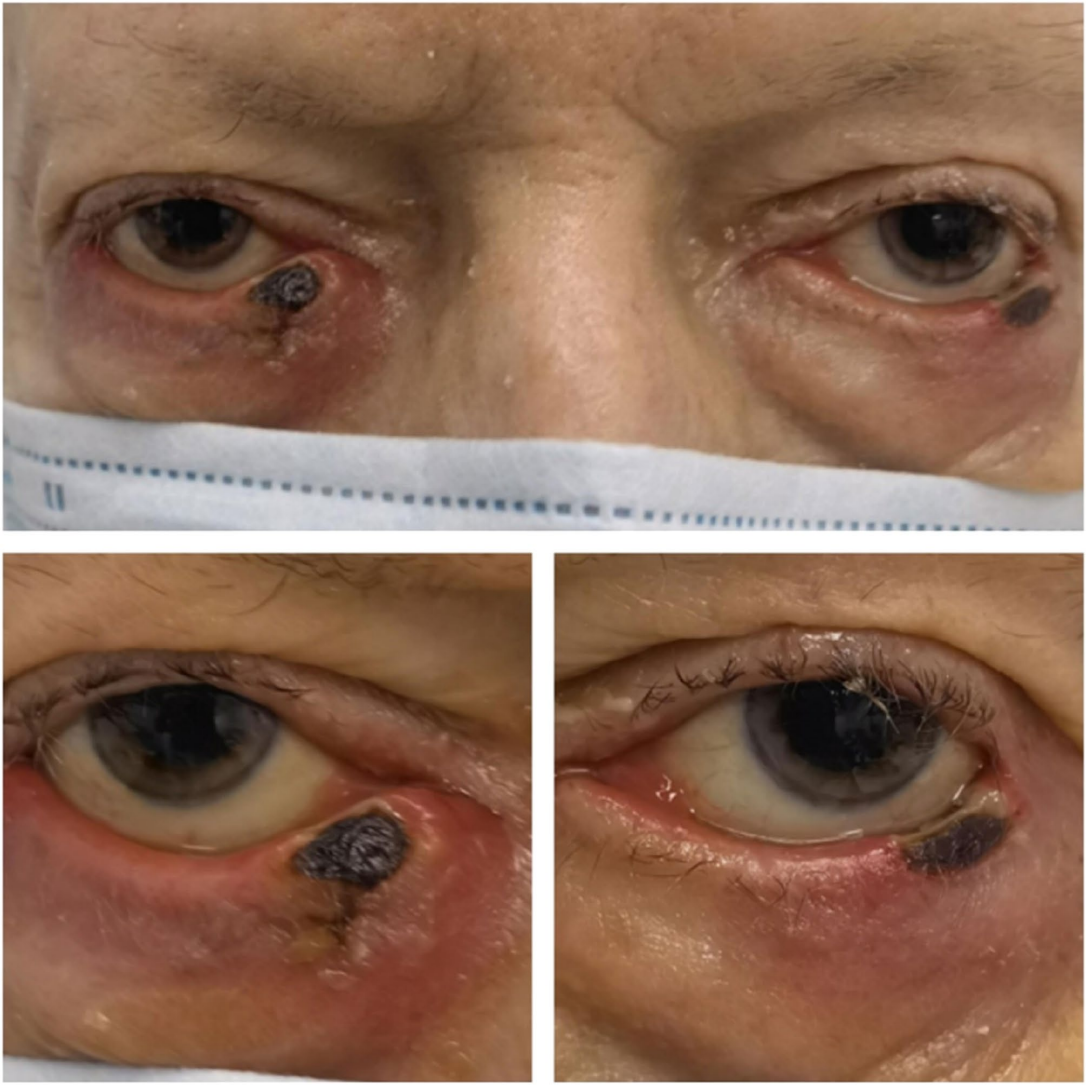
Necrotic lesions of both eyelids at the presentation

During hospitalization, he developed lesions on both lower eyelids, which gradually darkened in color over time. The ophthalmology department was consulted to assess the patient. The lesions appeared necrotic, irregular, non-ulcerated, and non-mobilizable, accompanied by whitish secretions (Fig. 1). Additionally, there was inflammation in the conjunctiva, while the cornea remained clear, and the anterior chamber was without inflammation in both eyes.

Systemic treatment with piperacillin-tazobactam 4.5 g IV every 6 h was started. Local treatment was added, with vitamin A ointment, artificial tears, and topical tobramycin drops. Although biopsy was considered to rule out malignancy, in light of the patient’s low platelet count (28 G/L), we elected against it, maintaining close observation.

Our suspicion leaned towards gangrenous pre-septal cellulitis secondary to Pseudomonas aeruginosa (known in dermatology as ecthyma gangrenosum), especially considering his chronic pancytopenia (leukocytes 0.68 G/L, neutrophils 0.21 G/L). Conjunctival swabs confirmed the presence of Pseudomonas aeruginosa three days later.

**Fig. 2:**
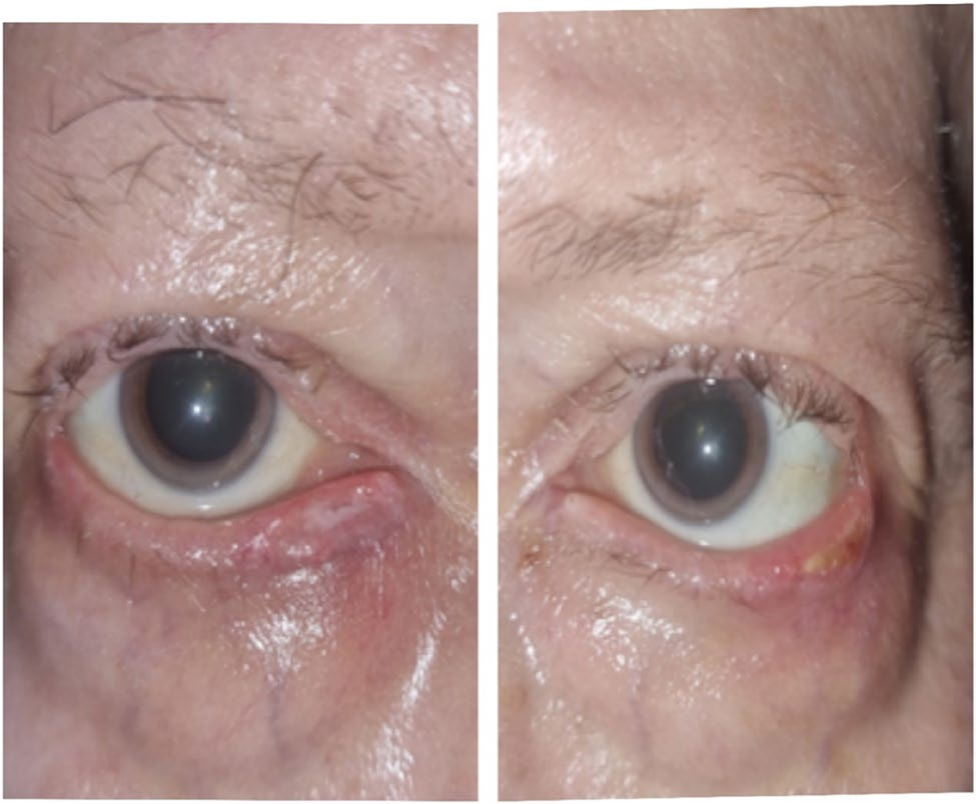
Evolution of the lesions of both eyelids

Two weeks later, the lesions had resolved, leaving only minimal skin sequelae (Fig. 2).

## Discussion

It is important to clarify that while previously published cases used terms such as ‘blepharitis’ or ‘blepharoconjunctivitis’, our diagnosis is better classified as pre-septal cellulitis based on the clinical presentation and localization of the lesions.

Blepharoconjunctivitis represents a common affliction among individuals with dermatitis and dry skin conditions [1]. Bacterial infections frequently exacerbate this condition, particularly by pathogens such as *Streptococcus* species and *Staphylococcus* aureus. However, with the advent of a wide array of antibiotics, these bacteria have diminished in significance, particularly for patients with compromised immune systems due to chemotherapies or haematological conditions. Instead, individuals in this vulnerable population face heightened susceptibility to *Pseudomonas aeruginosa*, which can lead to more severe and deleterious consequences [3–5]. A literature search revealed only eight case reports of *pseudomonas aeruginosa* induced blepharitis [1, 6–12]. One case involved a patient experiencing leukopenia induced by chemotherapy for pulmonary metastases from her breast cancer [10]. She presented with eyelid swelling and erythema, with a positive culture for *Pseudomonas aeruginosa*, which rapidly progressed to gangrenous lesions. Treatment with systemic and topical antibiotics was successful, although it resulted in a prominent residual defect. In 1985, another case was reported in Germany, involving a newborn boy who presented with severe bilateral necrotizing blepharitis secondary to transient neutropenia [7]. Unfortunately, this condition resulted in the loss of vision in his right eye. Three years later, another case involved a 16-month-old infant who presented with acute lateral cantholysis and eyelid necrosis secondary to severe *Pseudomonas aeruginosa* blepharitis and preseptal cellulitis, occurring in the setting of malnutrition and failure to thrive [9]. The lesions underwent satisfactory self-healing with the institution of antibiotic therapy and proper diet. More recently, in 2017, a 10-month-old child was reported with ecthyma gangrenosum affecting the periorbital region [11], caused by *Pseudomonas aeruginosa*. The infant initially presented with fever, eyelid swelling, and erythema, which progressed to necrosis. Successful treatment was achieved with aggressive antibiotic therapy, immunoglobulin administration, and surgical debridement. Another case involved a patient who was believed to have been infected iatrogenically via contaminated shampoo that was being used as a preventive measure against recurrent blepharoconjunctivitis [1]. The infection was treated with topical antibiotics, resulting in rapid recovery. In 1996, a case of *Pseudomonas aeruginosa* blepharitis in a patient with vancomycin-induced neutropenia was reported in Canada. Treatment involved both systemic and topical antibiotics, which were successful in treating the infection [8]. However, despite the patient’s neutrophil count recovery and survival from the infection, radical reconstructive surgery of his eyelids was required. Another case was reported in 1997, involving a patient who received consolidation chemotherapy for acute lymphocytic leukemia acquired necrotizing *Pseudomonas aeruginosa* blepharoconjunctivitis of the right eye during a period of mild leukopenia [6]. The infection led to severe orbital and periorbital inflammation, which extended down to the neck. High-dose antibiotic treatment with ceftazidime and tobramycin combined with granulocyte cell-stimulating factor cleared the infection after several days. However plastic surgery was required to restore normal eye closure. In 2009, A 60-year-old man with AIDS and a history of recurrent diffuse large B-cell lymphoma undergoing chemotherapy presented with purulent discharge from his left eye, with edema and erythema, but without fever. He was diagnosed with *Pseudomonas aeruginosa* preseptal cellulitis with localized necrosis (ecthyma gangrenosum) [12]. Within hours, the necrotic lesion and surrounding erythema rapidly worsened, showing improvement only after the initiation of antipseudomonal antibiotics. Unfortunately, despite initial signs of recovery, the patient died ten days later from pneumonia and sepsis caused by methicillin-resistant *Staphylococcus aureus* (MRSA) and *Enterococcus faecium*.

The condition is often associated with rapid necrosis of skin tissue, but this resolved with time with appropriate treatment.

Our patient developed gangrenous lesions likely due to both his underlying pre-septal cellulitis and the pancytopenia following chemotherapy. Fortunately, a combination of local and systemic treatment halted disease progression, leaving only mild cutaneous sequelae, which can be addressed later if necessary. He was discharged from the hospital without the need for oral antibiotics.

Patients with similar presentations need to be closely monitored, as any further necrosis would require extensive surgical debridement. Indeed, necrotizing fasciitis is a possible and extremely serious development for the patient.

## Conclusion

This report emphasizes the critical importance of considering ecthyma gangrenosum as a potential diagnosis in all immunocompromised patients presenting with cutaneous lesions, including erythema, redness, and pigmentary changes. While laboratory tests are valuable for confirming the diagnosis, they should not delay the initiation of treatment. Prompt diagnosis and intervention are paramount to prevent devastating damage, particularly to delicate areas such as the periorbital region. Early clinical recognition of this condition is crucial for timely management and improved patient outcomes. Clinicians should maintain a high index of suspicion and be prepared to commence treatment based on clinical presentation, even as confirmatory tests are pending. This approach ensures that potentially life or organ-saving interventions are not delayed, especially in cases where rapid progression of the disease could lead to severe complications.

## Data Availability

No datasets were generated or analysed during the current study.

## References

[1] Brook I, Hulbard C (1993) Pseudomonas aeruginosa iatrogenic blepharoconjunctivitis. Arch Ophthalmol 111(1):26 8424719 10.1001/archopht.1993.01090010028018

[2] Smolin G, Okumoto M (1977) Staphylococcal blepharitis. Arch Ophthalmol 95(5):812–816 324453 10.1001/archopht.1977.04450050090009

[3] Hughes WT (1989) Infections in children with malignant disease. In: Pizzo PA, Poplack DG (eds) Principles and practice of pediatric oncology, 1st edn. Lippincott, Philadelphia, pp 521–547

[4] Paprocka P, Durnaś B, Mańkowska A, Król G, Wollny T, Bucki R (2022) Pseudomonas aeruginosa infections in cancer patients. Pathogens 11(6):679 35745533 10.3390/pathogens11060679 PMC9230571

[5] Qin S, Xiao W, Zhou C, Pu Q, Deng X, Lan L et al (2022) Pseudomonas aeruginosa: pathogenesis, virulence factors, antibiotic resistance, interaction with host, technology advances and emerging therapeutics. Signal Transduct Target Ther 7(1):199 35752612 10.1038/s41392-022-01056-1 PMC9233671

[6] Giagounidis AAN, Meckenstock G, Flacke S, Burk M, Wehmeier A, Aul C et al (1997) Pseudomonas aeruginosa blepharoconjunctivitis during cytoreductive chemotherapy in a woman with acute lymphocytic leukemia. Ann Hematol 75(3):121–123 9368482 10.1007/s002770050325

[7] Huber-Spitzy V, Steinkogler F, Grabner G (1985) Nekrotisierende pseudomonasinfektion der lider und tränenwege Mit orbitaler beteiligung Bei einem Neugeborenen. Klin Monatsblätter Für Augenheilkd 187(10):287–289 10.1055/s-2008-1054314 3906251

[8] John MA, Austin TW, Bombassaro AM (1996) *Pseudomonas aeruginosa* blepharitis in a patient with Vancomycin induced neutropenia. Can J Infect Dis 7(1):63–65 22514419 10.1155/1996/301396 PMC3327369

[9] Prendiville KJ, Bath PE (1988) Lateral cantholysis and eyelid necrosis secondary to Pseudomonas aeruginosa. Br J Ophthalmol 72(4):292–294 3408085

[10] Rosenoff SH (1974) Pseudomonas blepharoconjunctivitis: a complication of combination chemotherapy. Arch Ophthalmol 91(6):490 4363858 10.1001/archopht.1974.03900060504016

[11] Lin Q, Hu B, Shi J, Shi W, Cao W, Li L (2017) Eyelid and periorbital ecthyma gangrenosum due to *Pseudomonas aeruginosa* in an infant. Pediatr Investig 1(1):47–49 32851218 10.1002/ped4.12005 PMC7331313

[12] Hulten EA, Shah AA, Petersen KN, Gallagher CM, Vangeertruyden PH, Kortepeter MG (2009) Pseudomonas aeruginosa preseptal cellulitis and focal necrosis in a patient with severe Immunocompromise. Infect Dis Clin Pract 17(5):346–348

